# Exploring the multidimensional relationships between social media support, social confidence, perceived media credibility and life attitude during the COVID-19 pandemic

**DOI:** 10.1007/s12144-022-03238-0

**Published:** 2022-05-25

**Authors:** Xiaoquan Pan, Yiqun Luo

**Affiliations:** Xingzhi College, Zhejiang Normal University, 688 Yingbin Road, Jinhua, Zhejiang Province People’s Republic of China

**Keywords:** Social media support, Social confidence, Perceived media credibility, Life attitude, COVID-19

## Abstract

Previous literature mainly focuses on the impact of social media support on social trust, emotional effect and life attitude, and affirmed the social governance value of social media support. However, the multidimensional relationship between social media support and social confidence, perceived media credibility and life attitude during the COVID-19 pandemic is an important, yet less explored, research issue. The present research aimed to fill in this gap by a survey of 1343 participants who are permanent residents aged 18 and above in a city through WeChat social networking platform. The results showed that: (1) social media support exerted direct influence on social confidence, perceived media credibility and life attitude; (2) perceived media credibility positively influenced social confidence; (3) social media support not only directly promoted social confidence, but also indirectly influenced social confidence through public’s perceived media credibility. These findings suggested that strengthening social media support during the COVID-19 pandemic is not only helpful to reconstitute the public’s confidence in prevailing against the pandemic, but also is able to help promote the diversification of the power of public network autonomy. This study highlighted social media support as an effective path to improve the ability of social governance.

## Introduction

The outbreak of novel coronavirus pneumonia 2019 (COVID-19), which was initially uncontrollable and unpredictable (Karataş & Tagay, 2021), has become a major public health emergency in the world. Under the centralized and unified leadership, the Chinese government has united and led the people to effectively control the epidemic of COVID-19, and provided experience and confidence for countries globally to combat the epidemic of COVID-19. As a novel coronavirus pneumonia epidemic characterized as high uncertainty and high infectious, and fatal characteristics (Ma et al., 2020), COVID-19 has seriously threatened the health of the people, and has also caused mass panic (Bacon & Corr, 2020). Besides, grounded on existing researches, many of the mental health symptoms are also ascribed to COVID-19, such as depression, anxiety, stress, post-traumatic symptoms (Xiong et al., 2020; Wang et al., 2020a, b, 2021a, b) and suicide (McIntyre et al., 2021). The inevitable mass panic and mental health symptoms primarily stem from the public’s worries and fears about the uncertainty of the epidemic information. What is missing in current literature is a conceptualization of how social media information works to affect the public’s social confidence and life attitude so as to alleviate mental health risks. Therefore, in response to the COVID-19 pandemic, we should not only probe into epidemic prevention and control and medical treatment, but also give prominence to the utility of social media to provide support to the public, including information release, psychological counseling and strategic guidance (Karataş & Tagay, 2021; Johnson & Kaye, 2015). Previous research emphasized the significance of the selection of appropriate communication channels for the dissemination of information to reach the population at risk (Zhang et al., 2014). Such researches identified that the most trustworthy sources of information during a crisis came from social media, such as newspaper, television, mass media, and Web-based sources (Wong & Sam, 2010; Bults et al., 2011; van Velsen et al., 2012). Effectively guiding public opinion and strengthening the confidence to overcome the epidemic situation is of great significance for the deepening and promotion of epidemic prevention, and is related to the construction of a healthy social mentality and the stability and development of national politics and economy as well. It is one of the important challenges that social governance is confronted with.

Under the background of COVID-19 facing the world, social media play a significant role in constructing a main keynote of grasping the issue, creating a harmonious public opinion environment and promoting the coordinated development of society. In the face of the transparency of information and the continuous increase of information flow in the network age, social media characterized as foresight, predictability and timeliness can meet the public’s right to know through organized and planned active reporting. On the one hand, social media assist people adopting positive life on the journey of facing the epidemic of COVID-19, with the help of the power of public opinion; On the other hand, social media can be utilized to do a good job in education, counseling and stability, and resolve emotions and accumulated grievances, thus making the general public generate the hope of overcoming the epidemic and enhance social confidence (Mo et al., 2014).

Current research literature has asserted the impact of social media support on social trust, emotional effect and life attitude, and affirmed the social governance value of social media support (Porumbescu, 2017; Zhao et al., 2016). For instance, previous study supported that social media use was positively associated with psychological well-being and negatively associated with social isolation (Choi & Noh, 2020). Besides, related study indicated that social media itself can also be used as a channel to combat rumors (Tripathy et al., 2013). However, the multidimensional relationship between social media support and these positive behaviors during the COVID-19 pandemic is an important, yet less explored, research issue. This study, grounded on social media support as the starting point, intends to explore and analyze the multidimensional relationship between social media support, social media credibility, social confidence and life attitude perceived by the public facing the epidemic of COVID-19 so as to provide psychological basis for advocating the public to consciously resist the COVID-19 and promoting more active public opinion guidance.

## Literature Review

### Social Media Support

Social media were defined as a new type of online media giving users great engagement venue, which share most or all of the following characteristics: participation, openness, conversation, community and connectedness (Mayfield, 2007). The definition of social media by communication scholars Kaplan and Haenlein (2010) is the most widely used in academic circles: Social media is a series of network applications based on Web2.0 technology and ideology, which allows users to create and communicate user-generated content (UGC). In addition, scholars thought that social media facilitate online information-sharing activities (Ransbotham & Kane, 2011), provide innovative socially mediated channels for information acquisitions and have thus become a significant venue of perceiving social support (Lin & Kishore, 2021). For instance, related empirical research indicated that social media enable adolescent users to strengthen bonds with existing friends and to form new friendships online, which reduce social isolation and loneliness, and indirectly improve mental health (O’Keeffe et al., 2011). From the above, the development of social media contributes to the establishment of interactive technology-based community, thus promoting social connectedness, information sharing and collaboration (Aral et al., 2013; Kaplan & Haenlein, 2010) and triggering user engagement on social media (Majchrzak et al., 2013). What distinguishes social media from traditional media is that it brings users a strong sense of social media affordances (Cabiddu et al., 2014), which meets the needs of users to establish relationships and exert influence (Volkoff & Strong, 2013). Research studies have depicted the concept of “social media” in accordance with that of “social network service”, but under China’s social circumstances, China’s social networks play more the role of social media. For instance, most users look through a large amount of information every day, but very little is really related to their own reality. Therefore, in this study, the word “social media” is adopted to highlight the social network sites from which social support is available (Lin et al., 2016).

The concept of social support originally stemmed from the field of presence and psychiatry. Considerable literature indicates that social support assists in improving patients’ health behaviors and health outcomes through self-management of chronic disease (Yan & Tan, 2014). While psychology and sociology brought this concept into their own system, social support commonly refers to “information leading the subject to believe that he is cared for and loved, esteemed, and a member of a network of mutual obligations”(Cobb, 1976, p.306), emphasizing the support system obtained by individuals from society that is perceptible, spiritual and instrumental and can help individuals solve problems and cope with crises. In light of existing literature of the impact of social media on developing social interactive relationships (Majchrzak et al., 2013; Cabiddu et al., 2014), social media support is mainly reflected in the following perspectives.

Firstly, the advanced technology-constructed social media provide convenient venue for people to independently obtain information and express selves. Meanwhile, the massive information transmitted through multiple channels in the network also exerts special requirements on individuals’ ability to understand and judge information. People’s experience in collecting, identifying and verifying information accumulated in offline life is obviously insufficient to help them cope with trust problems in the network environment, and the formed habits of network information dependence aggravate individuals’ difficulty in resisting the influence of social media, thus yielding negative emotions, cognitive conflicts and health-related questions (Genuis, 2013). In this case, social media should be effectively integrated into personalized life and realize the intersection of network society and public opinion with real social life, which is consistent with the previous research on the impact of social media on people’s life attitude (Bambina, 2007; Karahanna et al., 2018).

Secondly, social media information can eliminate uncertainty, resolve ambiguity and help build social confidence (Rettie, 2003). In the face of many events learned through the network, people expect to master more information close to the facts to help them make judgments/decisions because they are unable to be on the scene. With the help of social media platforms (Microblog, Wechat, forum, etc.), individuals conduct multidimensional and rapid exchange of views and opinions with others. As what Markus and Silver (2008) argued, “the continual emergence of new technologies inevitably requires ongoing conceptual development”(p.612),the technology-mediated environment enables individuals to not only make judgments/decisions based on emerging hot topics and multi-party views, but also affect others through the expression of their opinions, thus contributing to the development of social public opinion. As mentioned above, when the information resources obtained in the network environment are relatively scant to support personal cognitive judgment, individuals expect to pursue for informational, experiential and emotional support from a trusting social relationship (Lin & Kishore, 2021) and hope to stand with most people to resist risks by working together in the same boat (Wan et al., 2010). In such a state, collecting and processing information helps people understand their environment and situations, which is more important in the network society with complicated information and rapid changes in hot spots. In psychological research, the sense of control is used to describe the degree to which individuals control and influence their environment and are responsible for their own behavior (Ruthing et al., 2007). Research evidence has started to build up in support of the sense of control in enhancing people’s mental and physical health (Ruthing et al., 2007; Infurna et al., 2011). According to the theory of Dweck (2017), the sense of control stems from two factors: predictability and competence. The former is the need for people to understand the relationship between things in the environment. In view of the fact that it is unrealistic to fully grasp the social relationships, individuals anticipate to keep understanding new and complex situations. The latter is the survival skills developed by people to control the surrounding environment and even formulate or change rules, definitely demonstrating their personal initiative and achieving self-management (Barlow et al., 2002).

Thirdly, on the issue of network information trust, people’s perceived adequacy of information resources, controllability, authority, moralization and social consensus directly affect their predictability and competence of information delivery trust, thereby influencing the result of information trust. Utilizing familiarized social media to obtain reliable information assists individuals achieving the optimal psychic outcomes of trust (Turner & Kelly, 2000). In the social media environment, the establishment of social norm consensus and the improvement of institutional constraints help to reduce the risk of uncertainty, which also reflects the role of social psychological resources in the network life (Johnson & Kaye, 2015). With the promotion of people’s internal security motivation and the demand for accurate perceptual judgment, people dramatically demonstrate their thirst for the credibility of social media and social system security, thus triggering the formation and development of the network contract society.

### Impact of Social Media Support on Social Confidence, Perceived Media Credibility and Life Attitude

Social confidence, also known as public confidence, is depicted as a psychological force which makes the public believe that a certain thing (goal) can be achieved in the future, reflecting the public’s recognition and trust in a certain action subject, a certain thing and a specific object, as well as the stable psychological expectations formed on this basis (Martino et al., 2017; Delhey & Newton, 2003). As pointed out in the study of Catterson et al. (2015), social confidence is people’s expectation and judgment on the development situation of their society and individual development prospects on the basis of their real life experience. In the COVID-19 pandemic, social confidence is mainly manifested in the confidence to overcome the epidemic situation, that is, the public’s cognition and expectation of the overall epidemic situation and the impact on individuals. Research has shown that the public’s cognition and comprehensive evaluation of the government and social institutions are important factors affecting social confidence (Johnson & Kaye, 2015). As so, social confidence is characterized as an important reference index for individuals to plan and adjust strategies in the future (Catterson et al., 2015). According to Johnson and Kaye (2015), social confidence stems from citizens’ cognition and comprehensive evaluation of the current government performance, including social stability, policy rationality, government administrative effectiveness, and citizens’ participation in national and social public affairs. Some scholars proposed that different forms of social media will promote or dispel people’s trust in political institutions in their own ways and become a catalyst for political expression and democratic promotion (Hyun & Kim, 2015; Shao & Wang, 2017). Compared with traditional media, social media provide people with more personalized information and change people’s social cognition and behavior. In China’s unique cultural background, the significance of social media is also reflected in the fact that it brings a wide variety of relational information, which not only supports individuals’ beliefs or decision-makings, but also helps them effectively predict social development. More specifically, “given that relational information (e.g., social groups) is paramount in defining the self and understanding others in collectivistic cultures, it is likely that actors will seek and rely on this source of information to enhance confidence in social predictions” (Gelfand et al., 2015, p. 502). Although in the era of traditional media, media trust can promote public confidence in the government and its institutions (Gronke & Cook, 2007), in the era of social media, the conclusions of relevant studies are inconsistent. Some studies argued that the intervention of social media will make government affairs more open and transparent, and increase the public’s political participation, thereby improving the public’s social confidence (Porumbescu, 2017); On the contrary, there is current literature holding that social media has created a polarized public space in the political culture of our society (Shao & Wang, 2017), which may damage social confidence. This study believes that in the context of the epidemic of COVID-19, the support of social media can strengthen the public’s awareness of the pandemic, help stabilize the psychological state, and is expected to accelerate the construction of social confidence.

Media credibility is depicted as the capability of media to win public trust in their relationship with the public and whether or not information about source credibility is enough (Wierzbicki, 2018). Researches on media credibility have mainly laid stress on examining components to assess perceived media credibility with. As early as 1950s, Hovland and Weiss (1951) suggested trustworthiness and expertise as source credibility factors. From then on, related studies have suggested a few components that affect media credibility. These factors include expertise, dynamism, and trustworthiness (Whitehead, 1986), concern for community well-being and factual foundations of information published (Rimmer & Weaver, 1987), social concern and credibility of paper (Meyer, 1988), and composure and sociability (Gass & Seiter, 1999). There is existing study concluding that the credibility of social media can be enhanced in people’s continuous use (Wierzbicki, 2018). Additionally, scholars have found that the research on media credibility mainly focuses on two categories: information sources and communication channels (Kiousis, 2001; Christensen, 2011). The source of social media is primarily classified into official account and unofficial account. Official accounts involve those opened by governmental identity and official news media. In view of the mouthpiece role of news media in China, the social media accounts opened by news media usually represent the official voice. Unofficial accounts mainly refer to accounts opened by individuals on social media. This study highlights that in the COVID-19 pandemic, the relationship between social media trust and the public’ confidence in combating the epidemic relies on the authoritative information released by social media which helps the public construct rational judgment and reasonable expectation, and then improves the confidence in prevailing against the epidemic.

Attitude research began in social psychology with a far-reaching and complex history. Attitude was defined as related to the perspectives of cognition, emotion and intention respectively (Rokeach, 1952; Edwards et al., 1998). Enlightened by Edwards et al.’s definition of attitude, George and Park (2016) proposed that life attitude is an individual’s emotional response to his/her life on the basis of self-determined standards. Similarly, life attitude has been identified as a complete psychological reaction process composed of needs, wishes, psychological tendencies, values, specific views and emotions existing in the mind and expressed through behavior (Heintzelman & King, 2014). The influence of life attitude on individuals has been confirmed by many scholars. Michael and Charles (1992) asserted that optimistic life attitude will affect people’s life satisfaction and subjective well-being, thus constituting one of the indicators to predict life satisfaction. The research of Chang (2002) found that a positive and optimistic attitude is of great significance to reduce psychological problems and obtain higher life satisfaction. In light of Uznaze’s set theory, Soviet psychologist Petrovski put forward the hierarchy theory, highlighting that behavior was determined by two factors: the needs of the subject and the corresponding objective environment, and stressing that the personal value orientation system reflected the individual’s life attitude towards economic, social, political and ideological principles. Therefore, life attitude can be used to illustrate the intuitive reflection of individual satisfaction with their own environment, that is, what we usually call attitude determines needs and behavior. Erwin et al. (2004) examined the role of social network support in alleviating people’s social anxiety. Likewise, Some studies have highlighted life attitude as an individually-based psychological construction which helps scaffold positive behavioral direction for the future and combat negative situations that challenge individual’s expectation value (Yek et al., 2017; George & Park, 2016). Studies support that those with low social support are more likely to suffer from mental health problems (Klineberg et al., 2006; Maulik et al., 2011). Related research also found an inverse correlation between supportive online interaction on social media and both depression and anxiety (Seabrook et al., 2016). Therefore, this study attempted to examine the underlying relationship between perceived social media support and life attitude, and whether participating in social media interaction potentially has an impact on the life attitude of social groups.

### The Present Study Hypotheses

**Fig. 1:**
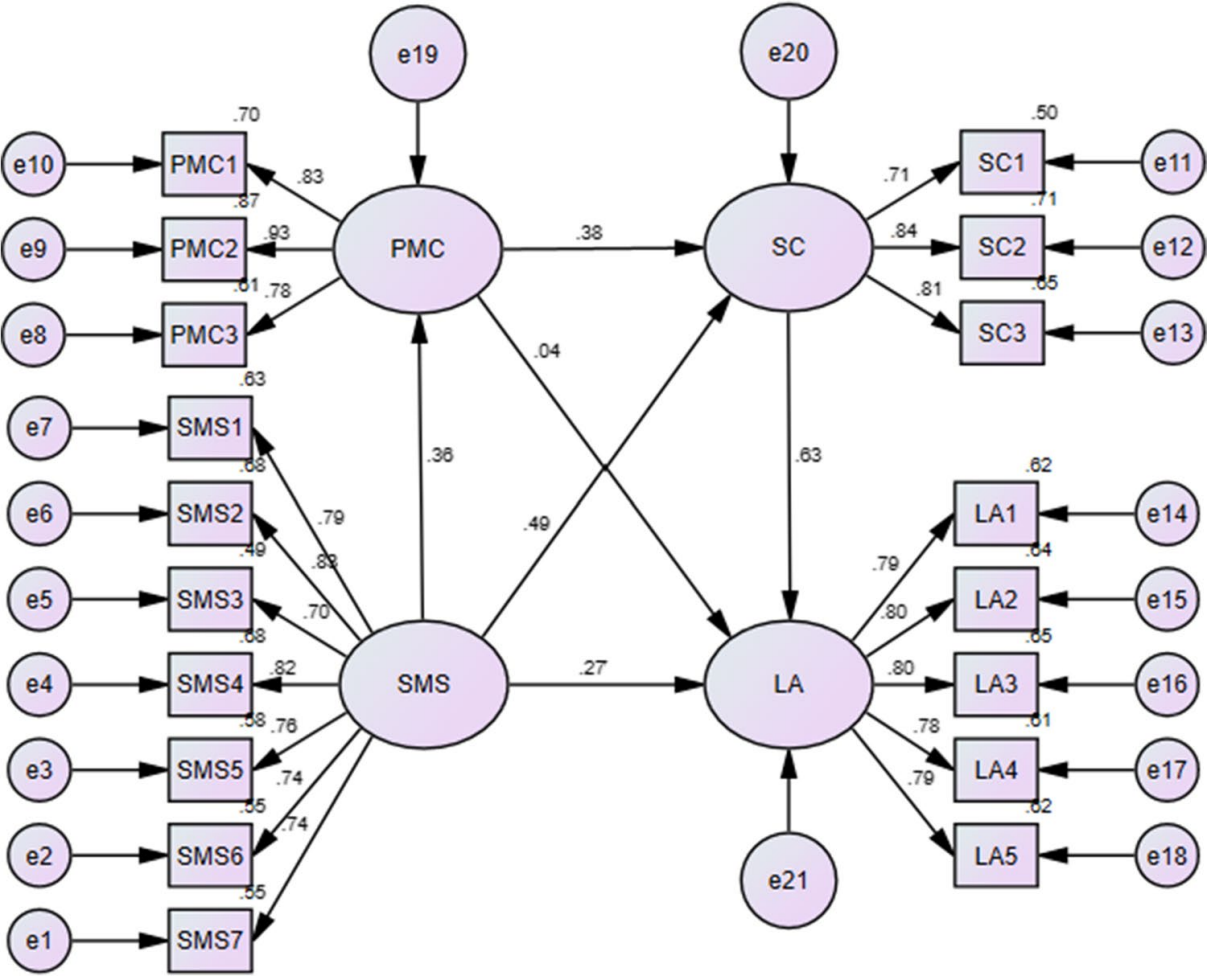
The Test Result of the Structural Model

The present study aimed to clarify the multidimensional relationship between social media support, social confidence, perceived media credibility and life attitude during the period of COVID-19. The relationships among the above variables were encapsulated in the research structural model (see Fig. 1). Although social media support, social confidence, perceived media credibility and their predictors/influential effects have been respectively examined in previous researches, Fig. 1 embodied the constructed associations from a newly-built structural model. Thereby, the following hypotheses were formulated.

- H1: Social media support significantly and positively influences social confidence.
- H2: Social media support significantly and positively influences life attitude.
- H3: Social media support significantly and positively influences perceived media credibility.
- H4: Social confidence significantly and positively influences life attitude.
- H5: Perceived media credibility significantly and positively influences social confidence.
- H6: Perceived media credibility significantly and positively influences life attitude.
- H7: Perceived media credibility significantly mediates the relationship between social media support and social confidence.
- H8: Social confidence significantly mediates the relationship between social media support and life attitude.
- H9: Social confidence significantly mediates the relationship between perceived media credibility and life attitude.

In this study, social media support was used as the determinant because of its close link to the public’s psychological state and subjective experience of social media value under COVID-19 circumstances. This study echoed the previous researches which emphasized the role mechanism of social media in social activities such as political participation and marketing.

## Methodology

### Participants and Procedures

Participants who are permanent residents aged 18 and above in a city were recruited through WeChat social networking platform (including personal WeChat friends circle and official account of enterprise). Totally, 1352 questionnaires were collected and after discarding incomplete ones, 1343 questionnaires are valid. Among the 1343 effective respondents, the average age was 35.2 years (SD = 9.35); Among them, men accounted for 40.21% (540 persons) and women accounted for 59.79% (803); 30.90% (415) had a monthly income of less than 10,000 yuan, 46.91% (630) had a monthly income of 10,000 ~ 15,000 yuan, and 22.19% (298) had a monthly income of more than 15,000 yuan; College degree or below accounted for 18.39% (247), undergraduate degree accounted for 64.11% (861), and graduate degree accounted for 17.50% (235); Students accounted for 10.50% (141), public institution staff accounted for 32.61% (438), and other occupations accounted for 56.89% (764). Participants all reported the behavior of using social media to get information about COVID-19 and social response.

### Measures

#### Social Media Support

Grounded on the expectation for which the public use social media to obtain information during the period of COVID-19, this study referred to the existing research results on individual perceived value of service (Sweeney and Soutar, 2001; Petrick, 2002), especially on the perceived support system of various social media, and thus divided the social media support scale into 7 items, including information support, emotional support and social support. One sample items is “Social media can give me a lot of useful information about COVID-19 combating”. On a six-point scale ranging from 1 (completely disagree) to 6 (completely agree), participants indicated the extent to which they agree with each of the statements. The ratings were averaged to index perceived social media support, with a higher score indicating a stronger social media support. Cronbach’s alpha was 0.91, indicating high reliability.

#### Social Confidence

A three-item social confidence scale revised from Michele et al. (2015) was adopted to measure the degree to which participants are confident of reconstructing normal social ecology. A sample item was “I think we can recover social economic development”. Participants were asked to rate each item on a six-point scale ranging from 1 (completely disagree) to 6 (completely agree), with a higher score indicating a greater level of social confidence. In this scale, Cronbach’s alpha was 0.83, indicating good reliability.

#### Life Attitude

A five-item life attitude scale adapted from the research of Steger et al. (2006) was used to measure the public’s life attitude. A sample item was “I can be myself in coping with the current difficulties”. Items were rated on a 6-point scale ranging from 1 (completely disagree) to 6 (completely agree), with a higher score indicating a higher level of life attitude. Cronbach’s alpha for this scale section was 0.90, indicating good reliability.

#### Perceived Media Credibility

The scale of perceived media credibility was developed and adapted from the previous published works of Gaziano and McGrath (1986) and Kiousis (2001), involving 3 items. A sample item was “I think the information released by official media accords with the reality”. Participants responded on a 6-point scale ranging from 1 (completely disagree) to 6 (completely agree). A higher score indicated a higher level of perceived media credibility. As Cronbach’s alpha was 0.88, the scale had good reliability.

### Modeling and Analysis

Firstly, SPSS 21.0 was adopted to conduct descriptive statistics of the study constructs and examine reliability and validity. Then, data were analyzed using structural equation modeling (SEM) approach to evaluate and modify the hypothesized conceptual model. The data were analyzed using Amos 21.0 with Maximum Likelihood Estimation to fit the model and estimate parameters. Following the recommendations by Hu and Bentler (1999), the model fit was tested by using several goodness-off it indexes, including the ratio of the chi-square to its degrees of freedom (X^2^/df), RMSEA, SRMR, CFI, and TLI. By Hair et al. (2010), values of X^2^/df(<3), CFI(>.90), TLI(>.90), RMSEA (<.08) and SRMR (<.08) are reflective of a good fit.

## Results

### Descriptive Statistics

The examining results of the mean, standard deviation of all the 18 items, Cronbach’s alpha values, and KMO and Bartlett’s Test were shown in Table 1. All mean scores were far above the mid-point of 3, indicating participants’ positive response to the variables in the questionnaire. The standard deviations ranged from 0.73 to 0.98, indicating a narrow spread of participants’ responses. Cronbach’s alpha values of the four constructs and results of KMO and Bartlett’s Test showed good reliability and validity.

**Table 1:** Descriptive Statistics of the Study Constructs

Constructs	Items	M	SD	Cronbach’s alpha	KMO and Bartlett’s Test
SMS	7	5.18	0.73	0.91	0.91^***^
SC	3	4.87	0.85	0.83	0.80^***^
LA	5	5.07	0.76	0.90	0.85^***^
PMC	3	4.66	0.98	0.88	0.81^***^

*SMS* Social Media Support, *SC* Social Confidence, *LA* Life Attitude, *PMC* Perceived Media Credibility. ***p < 0.001

### Test of the Structural Model

This study used Amos 21.0 with Maximum Likelihood Estimation to analyze the models and estimate parameters, including the procedures of assessing the reliability of items and variables, the convergent and discriminant validity, the path coefficients and the model predicative power. According to Teo and van Schaik (2012), “convergent validity, which examines whether individual indicators are indeed measuring the constructs they are purported to measure, was assessed using standardized indicator factor loadings, and they should be significant and exceed 0.7, and average variance extracted (AVE) by each construct should exceed the variance due to measurement error for that construct (i.e., AVE should exceed 0.50)” (p. 182). As can be seen, Table 2 indicated a good convergent validity of the structural model.

**Table 2:** Results of the structural model

Dimensions	SFL	S.E	t-value	CR	AVE (>0.50)
Social Media Support	0.70–0.83	0.081–0.104	12.72–15.28	0.91	0.59
Social Confidence	0.71–0.84	0.080–0.082	13.44–13.92	0.83	0.62
Life Attitude	0.78–0.80	0.063–0.068	15.29–16.33	0.89	0.63
Perceived Media Credibility	0.78–0.93	0.056–0.057	16.48–17.96	0.89	0.72

*SFL* Standardized Factor Loading, *S.E* Standardized Estimates, *AVE* Average Variance Extracted, *CR* Composite Reliability

Discriminant validity was measured by comparing the square root of the AVE for a given construct against the correlations of that construct with all other constructs (Henseler et al., 2015). Table 3 indicated that the square root of AVE (shown in parentheses along the diagonal) of each construct was higher (0.77 to 0.85) than corresponding correlation values for that variable in all cases, thereby assuring discriminant validity. As shown in Table 3, SC and LA had a relatively high correlation (r = 0.71), so collinearity variance inflation factors (VIFs) were calculated to examine potential multicollinearity problems. The VIF scores ranged between 1.96 and 4.35 (all<5), which indicated that the estimation of the regression coefficients would not be affected by multicollinearity problems (Montgomery et al., 2001).

**Table 3:** Discriminant validity for the measurement model

Constructs	SMS	SC	LA	PMC
SMS	(0.77)			
SC	0.55^**^	(0.79)		
LA	0.62^**^	0.71^**^	(0.79)	
PMC	0.32^**^	0.51^**^	0.44^**^	(0.85)

**p < 0.01. Diagonal in parentheses: square root of average variance extracted from observed variables (items); and off-diagonal: correlations between constructs. *SMS* Social Media Support, *SC* Social Confidence, *LA* Life Attitude, *PMC* Perceived Media Credibility

The structural model (see Fig.1) was tested using the whole dataset, and was found to have the following satisfactory model fit indices: X^2^/df = 2.82, CFI = 0.96, TLI = 0.95, RMSEA = 0.07, SRMR = 0.04.

As can be seen from Table 3, social media support was significantly and positively correlated with social confidence (r = 0.55, p < 0.01), life attitude (r = 0.62, p < 0.01), and perceived media credibility (r = 0.32, p < 0.01), thus preliminarily supporting Hypothesis 1, 2 and 3. Social confidence was significantly and positively linked to life attitude (r = 0.71, p < 0.01), thus preliminarily supporting Hypothesis 4. Moreover, perceived media credibility was significantly and positively associated with social confidence (r = 0.51, p < 0.01) and life attitude (r = 0.44, p < 0.01), thereby preliminarily supporting Hypothesis 5 and 6. To further examine the research hypotheses, the structural model analyses were conducted to be linked with the above correlations of variables. Figure 1 indicated that social media support has a significant predictive effect on social confidence (β = 0.49, p < 0.001), life attitude (β = 0.27, p < 0.001), and perceived media credibility (β = 0.36, p < 0.001), verifying and supporting Hypothesis 1, 2 and 3. Moreover, consistent with Hypothesis 4, social confidence has a significant predictive effect on life attitude (β = 0.63, p < 0.001). In addition, perceived media credibility has a significant effect on social confidence (β = 0.38, p < 0.001), supporting Hypothesis 5, but has no significant effect on life attitude (β = 0.04, p > 0.05), negating Hypothesis 6. A summary of the hypotheses testing results (H1-H6) was shown in Table 4.

**Table 4:** Hypothesis testing results (H1-H6)

Hypotheses	Path	Path coefficient	t-value	Results
H1	SMS-SC	0.49	7.94^***^	Supported
H2	SMS-LA	0.27	4.71^***^	Supported
H3	SMS-PMC	0.36	5.86^***^	Supported
H4	SC--LA	0.63	7.97^***^	Supported
H5	PMC--SC	0.38	6.35^***^	Supported
H6	PMC--LA	0.04	0.80	Not supported

^***^p < 0.001

### Mediational Analysis

From Fig. 1, social confidence mediated the relationship between social media support and life attitude and the relationship between perceived media credibility and life attitude. In addition, perceived media credibility also acted as mediator variable in explaining the relationship between social media support and social confidence. Therefore, the mediation effect was detected using bootstrapping test with structural equation model (Cheung & Lau, 2008). The results of the mediation analysis shown in Table 5 indicated statistically significance and accorded with the guidelines by Cohen (1988) with medium (0.1 < 0.5) indirect effect values, supporting Hypothesis 7, 8 and 9.

**Table 5:** Results of the mediational analysis (H7-H9)

From	β	Mediator	β	To	Indirect effect	95% confidence interval
SMS	0.36	PMC	0.38	SC	0.13^***^	[0.08–0.21]
SMS	0.49	SC	0.63	LA	0.41^***^	[0.32–0.52]
PMC	0.38	SC	0.63	LA	0.24^**^	[0.15–0.36]

^**^p < 0.001

## Discussion

This study developed a model to test the multidimensional relationships between social media support, social confidence, perceived media credibility and life attitude during the COVID-19 pandemic, and primarily examined the influence effect of social media support.

From the results, social media support exerted direct influence on social confidence, perceived media credibility and life attitude. This result echoed Wang et al. (2020b)‘s research which highlighted that “providing proper and repeated, yet simple, health education via the Internet and media is important for inculcating good hygiene practices”(p. 47) and that “perhaps, increased use of television (with participation by celebrities) and Internet (for detailed information with visual graphics and videos) to disseminate important health information might be more effective methods to change knowledge, attitude and practices among the general public” (p. 47). This result also demonstrated the significance of subjective perception of obtainable social support (Barrera, 1986) and meanwhile corroborated the previous researches which regarded perceived social support as being responsive to individuals’ needs (Feeney & Collins, 2015; Maisel & Gable, 2009). As highlighted in the recovery model (Needles & Abramson, 1990), perceived social support assisted people in redefining life situations and attribute positive life events to stable factors that could make people construct social confidence. Under the COVID-19 pandemic circumstances, the subjectively perceived social support, to a great extent, derives from the information released by social media. Firstly, through media support, people make expectations and judgments about the development situation of the society. The social confidence brought about by the public’s cognition and expectation of eventually prevailing against the pandemic will encourage individuals to take positive action and help reduce negative psychological symptoms (Duan & Zhu, 2020). Previous research indicated that the public’s cognition and comprehensive evaluation of the government are important factors affecting social confidence (Johnson & Kaye, 2015). Secondly, this study indicated a significant and positive influence of social media support on perceived media credibility. Media credibility is typically conceptualized as the ability of media to win public trust in their relationship with the public (Wierzbicki, 2018). While Kiousis (2001) decomposed the credibility construct into two components: information sources and communication channels, these perspectives are also applicable to the analysis of social trust. In China, official media and non-official media are two important information sources for the public to obtain information (Shao & Wang, 2017). With the advent of the new media era, on the one hand, individuals open accounts in social media to express their views or transmit information, and non-official media have become the representative of civil public opinion. On the other hand, traditional media and government agencies at all levels representing the main body of official public opinion set up accounts on social media platforms such as Microblog and Wechat. Therefore, social media have gradually become the communication channel of official information (Hyun & Kim, 2015). This study confirmed that social media support could help enhance individuals’ perceptions of media credibility, indicating that official social network media can not only spread authoritative news and major social information, but also play an important instructional role in the public’s awareness of the pandemic. Thus, official social media as well as non-official social media need to be normalized as key elements to stabilize social public opinion and social mentality. Thirdly, this research result demonstrated that social media support has a positive predicative effect on life attitude. This study held that in the COVID-19 pandemic, social media support contributes to the public’s trust in the official media, ensures the public’s attention to the authoritative information related to the pandemic, and helps the public form a rational judgment and reasonable expectation of the pandemic, so as to improve the confidence to overcome the epidemic and promote a positive attitude towards life.

This study found that perceived media credibility positively influenced social confidence. This is consistent with the research from Gronke and Cook (2007) which highlighted that people’s trust in the media can promote their confidence in government and other institutions. On a similar note, Porumbescu (2017) proposed that social media intervention made government affairs more transparent, shortened the time for the government to respond to people’s demands, and endowed people with more opportunities to participate in social management, which assists people in establishing higher trust on the government and social institutions and reconstructing social confidence. In addition, this study found that social confidence positively influenced life attitude. This echoed the previous research which indicated that social confidence as an individually-based psychological construction could help reduce negative emotions while improving psychological resilience, happiness, and life satisfaction (Yek et al., 2017).

This study further found that social media support not only directly promoted social confidence, but also indirectly influenced social confidence through public’s perceived media credibility. Johnson and Kaye (2015) held that social confidence stems from individuals’ cognition and comprehensive evaluation of the current government performance, including social stability, policy rationality, government execution and effectiveness, and citizens’ participation in national and social public affairs. Some scholars pointed out that different forms of social media (e.g., blogs, websites) will promote or dispel people’s trust in political institutions in their own ways, and have indeed become a catalyst for political expression and democratic promotion (Dimitrova et al., 2014; Krueger & Malecková, 2009), providing people with more personalized information and changing people’s social cognition and behavior. In China, the importance of social media is also reflected in the fact that it brings all kinds of relational information. The social media credibility can be enhanced by people’s continuous use of existing social media. In China’s unique cultural background, relational information can not only support individuals to believe or make certain judgments, but also help them effectively predict social development (Gelfand et al., 2015). On the other hand, social media support not only directly promoted life attitude, but also indirectly influenced life attitude through social confidence. Social confidence is the synthesis of social information and life attitude, otherwise it will produce negative rumors that will damage social confidence. To effectively deal with rumors, we should not only ensure the objectivity and authority of the information disseminated by the official media, but also lay stress on the public’s cognitive psychology and attitude towards the development of the epidemic. The traditional rumor governance mainly adopts the government leadership and united governance (Porumbescu, 2017), but this model has demonstrated a measure of malpractices, such as the rebounding of public opinion, the doubt of public credibility, etc. Therefore, paying attention to multiple social forces, especially the autonomy model of public participation, has gradually become a major breakthrough in building social confidence. The results of this study suggested that timely and effective information released by official media provides informational support to the public and incarnates the following functions: 1) improving the public’s confidence in overcoming the epidemic; 2) promoting the public to actively verify suspicious information; 3) encouraging the public to explore the truth of the incident and take the initiative to refute rumors. These are the key factors to promote the public to eliminate rumors and build social confidence, which is of great significance in building a self-confident, rational and positive social mentality. Noticeably, this study found that perceived media credibility had no direct influence on life attitude but indirectly influenced life attitude through the mediation of social confidence. This finding highlights the critical role of social confidence construction in decreasing the negative cognition (Abramson et al., 1989), and therefore singles out the importance of social confidence cultivation as an intermediary that transforms the effect of perceived social credibility into a psychological cognition, motivation for future life, and finally, recovery from hopelessness under COVID-19 (Zuo et al., 2021).

## Conclusion

This study explored multidimensional relationships between social media support, social confidence, perceived media credibility and life attitude during the COVID-19 pandemic. Most notably, the study findings indicated that social media support significantly and positively influenced social confidence, life attitude, and perceived media credibility. Second, social confidence positively predicted life attitude and acted as an intermediary between perceived media support and life attitude. Third, perceived media credibility was positively associated with social confidence and also mediated the relationship between social media support and social confidence. As such, the present study also theoretically contributed to establishing a solid foundation for the multidimensional relationship model of social media support in influencing the public’s confidence recovery during the COVID-19 pandemic.

Under the pandemic, social media support can not only directly promote the public’s perceived trust in information and information channels, but also has an important social impact by enhancing the public’s confidence in overcoming the epidemic. Therefore, strengthening the “timely, accurate, open and transparent” attribute of social media coverage during the COVID-19 pandemic is not only helpful to reconstitute the public’s confidence in prevailing against the pandemic and winning the battlefield of epidemic prevention and control, but also is able to help promote the diversification of the power of public network autonomy. This study highlighted social media support as an effective path to improve the ability of social governance and continuously promote the modernization of the national governance system. Of course, this study has some limitations. First of all, the results of this study were grounded on a comparatively small sample, which may give rise to a potential bias that will affect the degree to which these results are generalisable. The future study may entail involving a larger sample to include different types of individuals. Secondly, the simplex cross-sectional design being applied in this study may result in a common method bias. Hence, it is suggested that future study adopt multi-layered, muti-dimensional methods (e.g., the combination of cross-sectional design with qualitative research) to enhance our understanding of the causality as far as possible. Thirdly, the controlling variable of gender was not incorporated in this study. Future studies are needed to examine the measurement invariances of the SEM model with the controlling variable of gender and to test the potential effect of gender. Fourth, this study measured a small cohort of people in China, and thus the applicability of its findings may be limited. However, the key findings in this model are largely consistent with studies on the global COVID-19 public health crisis in different cultures (McIntyre et al., 2021; Wang et al., 2021a; Satici et al., 2020a). For instance, rumination fed by uncertainty may negatively affect wellbeing through fear of COVID-19, while the media plays an important role in the dissemination of information in the pandemic (Satici et al., 2020b). Fifth, in surveying the variable of social media support, the designed items did not distinguish the official media and non-official social media, therefore, future studies are suggested to further differentiate the official and non-official social media sources in order to compared their influences on the public’s social psychological cognition.

## Data Availability

The datasets generated during and/or analyzed during the current study are available from the corresponding author on reasonable request.
